# Babesiosis in Long Island: review of 62 cases focusing on treatment with azithromycin and atovaquone

**DOI:** 10.1186/s12941-017-0198-9

**Published:** 2017-04-11

**Authors:** Ekaterina A. Kletsova, Eric D. Spitzer, Bettina C. Fries, Luis A. Marcos

**Affiliations:** 1 Department of Medicine, Division of Infectious Diseases, Stony Brook University, Stony Brook, USA; 2 Department of Pathology, Stony Brook University, Stony Brook, NY USA; 3 Global Health Institute, Stony Brook University, Stony Brook, NY USA

**Keywords:** Babesiosis, Tick-borne, Babesia, Azithromycin, Atovaquone

## Abstract

**Background:**

Babesiosis is a potentially life-threatening, tick-borne infection endemic in New York. The purpose of this study was to review recent trends in babesiosis management and outcomes focusing on patients, who were treated with combination of azithromycin and atovaquone.

**Methods:**

A retrospective chart review of patients seen at Stony Brook University Hospital between 2008 and 2014 with peripheral blood smears positive for *Babesia* was performed. Clinical and epidemiological information was recorded and analyzed.

**Results:**

62 patients had confirmed babesiosis (presence of parasitemia). Forty six patients (74%) were treated exclusively with combination of azithromycin and atovaquone; 40 (87%) of these patients were hospitalized, 11 (28%) were admitted to Intensive Care Unit (ICU), 1 (2%) died. Majority of patients presented febrile with median temperature 38.5 °C. Median peak parasitemia among all patients was 1.3%, and median parasitemia among patients admitted to ICU was 5.0%. Six patients (15%) required exchange transfusion. Majority of patients (98%) improved and were discharged from hospital or clinic.

**Conclusion:**

Symptomatic babesiosis is still rare even in endemic regions. Recommended treatment regimen is well tolerated and effective. Compared to historical controls we observed a lower overall mortality.

## Background

Tick-bone infections are common in some geographic areas of the United States—mainly northeastern regions particularly in New York, Massachusetts, Rhode Island, and Connecticut [1–5]. Babesiosis is an emerging, usually tick-mediated infection caused by intra-erythrocytic parasites that may also be transmitted by blood transfusions [6].

The severity of Babesiosis ranges from asymptomatic or mild, self-limited febrile illness [5, 7] to potentially life threatening infection and may have a complicated clinical course especially in people with certain risk factors such as those with splenectomy, cancer, human immunodeficiency virus infection (HIV), chronic heart, lung, or live disease, or patients receiving immunosuppressive therapy [8]. A trend of increasing frequency of transfusion mediated babesiosis since the early 2000s was noted in recent studies [6, 9].

The combination of clindamycin and quinine was the first regimen of choice for the treatment of *Babesia microti* infection [8]. A combination of atovaquone and azithromycin is now recommended for mild to moderate disease [10] since it was shown that this combination is as effective as the combination of clindamycin and quinine but is associated with fewer adverse reactions. Clindamycin plus quinine is still recommended for patients with severe babesiosis, including those who require an exchange transfusion, as these patients were excluded from the azithromycin/atovaquone trial [11].

The purpose of this study was to review demographic, clinical characteristics, and outcomes of patients with proven babesiosis, who were treated with the combination of azithromycin and atovaquone.

## Methods

### Case definition

Laboratory records were reviewed to identify all adult patients who had a positive peripheral blood smear at Stony Brook University Hospital (SBUH) between 2008 and 2014. All initial positive smears were confirmed to be positive for *B. microti* by PCR analysis performed at the NY State Department of Health. Only symptomatic patients who had at least one positive smear were included in the study. These criteria met the definition of active babesiosis used in the current IDSA guidelines [10].

### Study design

A retrospective chart review was performed to gather descriptive clinical and epidemiological information. Only patients who were treated with combination of azithromycin and atovaquone were included into the analysis. Patients’ demographic information, past medical history, laboratory values, and outcomes were recorded and analyzed. SAPS II scores were calculated as previously described [12].

### Statistical analysis

Microsoft EXCEL was used to record and analyze the data. Proportions were calculated for all categorical variables and medians with interquartile ranges (IQR) were calculated for continuous variables.

## Results

### Number of cases

**Fig. 1:**
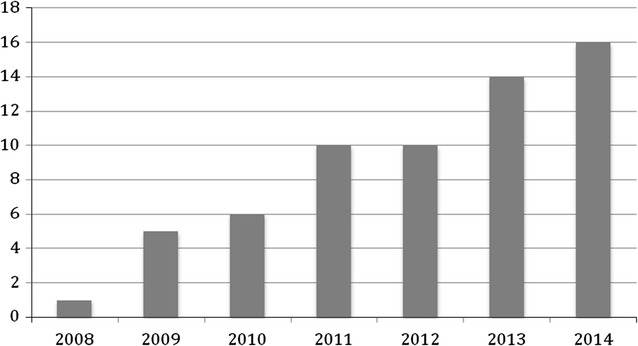
Number of patients admitted to SBUH with confirmed babesiosis by year

Between 2008 and 2014 a total of 62 patients presented to SBUH with active babesiosis confirmed by the presence of parasitemia on a peripheral blood smear. The incidence of babesiosis had trended up over the years from 1 case of an active babesiosis diagnosed in 2008 to 16 cases diagnosed in 2014 (Fig. 1). Fifty-three of the patients (85%) who presented with active babesiosis were admitted to the hospital. Twenty-seven of these patients (44%) were transferred from other hospitals in eastern Long Island with severe babesiosis for potential exchange transfusion; however, only 10 of these patients (38%) ultimately required exchange transfusion based on percent parasitemia and clinical findings.

### Treatment and ICU stay

Forty six patients (74%), who presented with active babesiosis, were treated exclusively with combination of atovaquone and azithromycin and were included into the analysis. Forty of the included patients (87%) were admitted to the hospital and 11 of the hospitalized patients (28%) required the intensive care unit (ICU) admission. The decision regarding ICU admission and management at the highest level of care was made based on clinical presentation as well as severity and number of comorbidities that increase risk for potential complication of babesiosis.

### Demographics and comorbidities

The median age of all admitted patients was 64 years (IQR 47–81 years). Twenty six patients (65%) were males (Table 1). Only 5 patients (13%) had a prior splenectomy but 4 (10%) had a history of malignancy, 17 (43%) had hypertension, 9 (23%) had a history of heart disease and some had multiple comorbidities (Table 1).

**Table 1:** Demographic and clinical characteristics of the patients treated with the combination of azithromycin and atovaquone

Characteristic	All (N = 40)	Admitted to ICU (n = 11)	Not admitted to ICU (n = 29)	P value
Age	64 (47–81)	60 (47–73)	66 (52–80)	0.44
Gender				
Male	26 (65)	8 (73)	18 (62)	0.58
Female	14 (35)	3 (27)	11 (38)	0.72
Race				
White	24 (60)	8 (73)	16 (55)	0.39
African American	0	0	0	
Hispanic	8 (20)	3 (27)	5 (17)	0.74
Asian	1 (3)	0 (0)	1 (3)	
Declined	2 (5)	0 (0)	2 (7)	
Other	5 (13)	0 (0)	5 (17)	
Comorbidities				
Hypertension	17 (43)	6 (55)	11 (38)	0.49
Diabetes mellitus	7 (18)	6 (55)	1 (3)	0.33
Heart disease (CHF/CAD/Arrhythmias)	9 (23)	3 (27)	6 (21)	0.84
Blood disease	2 (5)	0	2 (7)	
Cancer	4 (10)	0	4 (14)	
CKD	3 (8)	1 (9)	2 (7)	
COPD/asthma	6 (15)	2 (18)	4 (14)	0.89
Liver disease	2 (5)	2 (18)	0	
Autoimmune disease	3 (8)	0	3 (10)	
HIV	1 (3)	0	1 (3)	
Splenectomy	5 (13)	1 (9)	4 (14)	
Days in hospital	5 (1–9)	6 (2–11)	5 (1–11)	0.55
Clinical/lab characteristics				
SAPS II score (points)	20 (14–26)	21 (10–32)	20 (14–26)	0.69
Temperature on admission (C)	38.5 (37.2–39.8)	37.2 (35.6–38.8)	38.6 (37.6–39.8)	0.03
Peak parasitemia (%)	1.3 (2.5–5.1)	5.0 (1.5–11.5)	1.1 (1.1–3.3)	0.003
Days of parasitemia (number)	4 (1.75–6.25)	4.5 (1.5–7.5)	4 (1.2–6.8)	0.70
Hemoglobin (g/dL)	10.7 (8.4–13.0)	10.0 (7.9–12.2)	11.1 (8.7–13.5)	0.33
Platelets (×10^3^/μL)	74 (8.3–138.8)	64 (1.8–128.8)	74 (8.0–139.0)	0.66
AST (U/L)	79 (13–146)	87 (29–146)	72 (2–142)	0.52
ALT (U/L)	59 (4–114)	63 (1–125)	46 (4–96)	0.33
Total bilirubin (mg/dL)	1.3 (0.8–1.8)	1.5 (0.4–2.6)	1.3 (0.8–1.8)	0.40
LDH (U/L)	605 (231–979)	719 (282–1157)	535 (208–862)	0.12
Haptoglobin (mg/dL)	7.4 (6.7–8.1)	7.2 (1.5–15.9)	7.4 (7.1–7.7)	0.66
Exchange transfusions				
No	34 (85)	6 (55)	28 (97)	0.002
Yes (one)	6 (15)	5 (45)	1 (3)	0.42
Outcome				
Improved and discharged	39 (98)	11 (100)	28 (97)	
Died	1 (2)	0	1 (3)	

Data are presented as median (IQR) or No. (%)

### Fever and parasitemia

The median temperature on admission among all patients was 38.5 °C (IQR 37.2–39.8 °C). Interestingly, the median temperature of patient, admitted to the ICU was lower than median body temperature of those, admitted to regular medical floor (Table 1). The median recorded peak parasitemia among all patients was 1.3% and the maximum parasitemia observed was 11%. The median peak parasitemia tended to be significantly higher in patients admitted to the ICU (Table 1). Calculated median SAPS II score for all admitted patient was 20 points (IQR 14–26 points) indicating that patients had an average 4% hospital mortality risk during their admission. The score was not significantly different among the groups.

### Laboratory findings

The majority of patients in our sample exhibited anemia (median hemoglobin 10.7 g/dL), thrombocytopenia (median platelets 74 × 10^3^/μL), and elevated liver function tests (median alanine aminotransferase (ALT) 59 U/L, median aspartate aminotransferase (AST) 79 U/L, median total bilirubin 1.3 mg/dL). Also, laboratory evidence of hemolysis (median lactate dehydrogenase 605 units/L and median haptoglobin 7.4 mg/dL) was common. Generally, laboratory parameters were more abnormal in patients admitted to the ICU.

### Exchange transfusion

Six patients (15%) required an exchange transfusion in addition to antibiotic therapy. Five of these patients were admitted to the ICU. One patient, who had an exchange transfusion started on the floor prior to being admitted to the ICU, developed pulmonary edema followed by cardiac arrest and died on day two of hospitalization.

In general, the combination of atovaquone and azithromycin was well tolerated and effective. All except one patient improved and were discharged from the hospital in stable condition.

## Discussion

Our data suggested that incidence of babesiosis is trending up over the years (Fig. 1) in Suffolk county that is consistent with previous studies [5]. We cannot exclude that increased awareness of the infection leading to the increased overall number of tested patients confound these numbers.

This study was performed at a referral center in Long Island, NY, where babesiosis is endemic. Compared to historical controls ([13, 14]; Table 2), fewer patients were transferred to SBUH from other facilities suggesting better understanding of the disease in the medical community. Most of the patients diagnosed with acute symptomatic babesiosis were older adults. This may reflect under-recognition of this infection in younger adults and/or the presence of comorbidities in older adults. Babesiosis may be missed on a routine CBC (complete blood count) and requires a specific order for blood smear examination or other diagnostic tests. In their prospective case-finding and serosurvey study of babesiosis on Block Island, Rhode Island, Krause et al. [5] observed that the number and duration of symptoms due to Babesia infection were similar in people 20–49 years of age versus those older than 50 years of age; however, the latter group was more likely to be hospitalized.

**Table 2:** Comparison to other studies

	SBUH, 62 cases review	UAlbany 139 cases review	SBUH 34 cases review
Methods	Chart review of patients with positive smears from 2008 to 2014	Hospital records of babesiosis in NYS for 11 years (1982–1993)	Records of SBUH and VA^a^ hospitalized patients for 13 years with positive blood smears
		Classified to have mild or severe (death, >2 weeks in hospital, ICU admission)	Controls with FUO^b^, negative blood smears, matched by age and sex
Transferred from other hospital	27 (44%)	NA	30 (88%)
Median age (years)	64	66	46
Mean hospital stay (days)	9.6	11.7	12.7
ICU admission	20 (38%)	35 (25.2%)	–
Splenectomy	9 (15%)	16 (11.5%)	11 (32%)
Mean hemoglobin (g/dL)	10.6	11.3	10
Mean platelets (×10^3^/μL)	86	102	92
Mean LDH (U/L)	742	572	–
Mean ALT (U/L)	66	–	99
Mean AST (U/L)	85	–	121
Mean parasitemia (%)	3.4%	–	7.4%
Mean peak parasitemia (%)	4.4%	–	7.6%
Max parasitemia	25%	–	30%
Mean days of parasitemia	6	–	8.5
Treatment	Clindamycin 15 (24%) Quinine 13 (21%) Azithromycin 59 (95%) Atovaquone 59 (95%)	Clindamycin 110 (79%) Quinine 106 (76%)	Clindamycin 33 (97.1%) Quinine 28 (82.3%) Azithromycin 2 (5.9%) Atovaquone 15 (44%)
Exchange transfusions	12 (19%)	6 (4.3%)	7 (20.6%)
Died	1 (2%)	9 (6.5%)	3 (8.8%)
Associations	High-grade parasitemia and: Malignancy Splenectomy LDH AST Total bilirubin	Severe disease and: Cardiac disease/murmur Splenectomy Alkaline phosphatase WBC Higher parasitemia	Complicated babesiosis and: Hemoglobin <10 g/dL Higher parasitemia

For the purpose of a comparison, all patients from SBUH with positive blood smear were included in this table regardless of antimicrobials that were used for babesiosis therapy (n = 62)

^a^VA–Veteran Affairs Hospital

^b^FUO–Fever of Unknown Origin

The combination of atovaquone and azithromycin is now widely used for the treatment of patients presenting with babesiosis. The randomized study by Krause et al. [11] that demonstrated the effectiveness of atovaquone plus azithromycin specifically excluded patients with evidence of life-threatening babesiosis (e.g. encephalopathy, shock, congestive heart failure, pulmonary edema, DIC, or renal) or those who required exchange transfusion or assisted ventilation [11]. The current IDSA Guidelines contain an AIII recommendation for quinine plus clindamycin along with red blood cells (RBC) exchange transfusion for patients with severe babesiosis, defined as high grade parasitemia (≥10%), significant hemolysis, or renal, hepatic, or pulmonary compromise. All patients in our report had a disease severe enough to be hospitalized and to be admitted to the ICU, therefore our retrospective study suggests that atovaquone plus azithromycin is often effective for patients with moderate to severe babesiosis, many of whom may be admitted to ICUs because of associated comorbidities. Perhaps good supportive care and exchange transfusions, when indicated, together with antibiotics play more crucial role in patients’ outcomes than antiparasitic medications alone.

Compared to historical controls we observed a lower mortality—2 vs. 6.5% from the study of 139 hospitalized patients with babesiosis ([13]; Table 2) and 2 vs. 8.8% from the study of 34 hospitalized patients with babesiosis ([14]; Table 2).

Even though our report is focused on babesiosis patients treated with combination of azithromycin and atovaquone, it is noteworthy to discuss those patients, who were treated otherwise. Two pregnant patients were treated solely with clindamycin and quinine and 11 patients received all four antibiotics (clindamycin, quinine, azithromycin, and atovaquone), however not simultaneously (Table 3). Only 2 out of 62 patients with confirmed babesiosis did not respond to azithromycin/atovaquone combination (patient 1 and patient 5 in Table 1) suggesting that this regimen may be a reasonable initial therapy even in patients with a severe babesiosis. Even though two more patient were switched from azithromycin atovaquone to quinine/clindamycin (patients 2 and 3 in Table 3), the decision was made based on percent parasitemia on admission, but not on clinical failure of initial regimen.

**Table 3:** Patients treated with clindamycin and quinine in addition to azithromycin and atovaquone

Patient	Initial therapy	Changes	Admitted to ICU	ID involved
1	Azithromycin/atovaquone	Changed to clindamycin/quinine on day #5 due to poor clinical response	Yes	Yes
2	Azithromycin/atovaquone	Changed to clindamycin/quinine on day #2 due to high % parasitemia on admission → changed back to azithromycin/atovaquone on day #4 due to QT prolongation	Yes	Yes
3	Azithromycin/atovaquone	Initial therapy at outside hospital; started on clindamycin/quinine on admission to SBUH	No	No
4	Clindamycin/quinine	Changed to azithromycin/atovaquone on day #2 per ID recommendations	Yes	Yes
5	Azithromycin/atovaquone	Initial therapy started at the outside hospital → patient developed respiratory failure, intubated, changed to clindamycin/quinine, and transferred to SBUH	Yes	No
6	Clindamycin/quinine	Initial therapy was changed to azithromycin/atovaquone on day #1 per ID recommendations	Yes	Yes
7	Clindamycin/quinine	ID recommended to change regimen to azithromycin/atovaquone on day #2, however antibiotics were changed to azithromycin/clindamycin per primary team due to lack of IV formulation of atovaquone → therapy changed to azithromycin/atovaquone on day #7	Yes	Yes
8	Clindamycin/quinine	Therapy changed to azithromycin/atovaquone on day #6	No	No
9	Clindamycin/quinine	Therapy changed to azithromycin/atovaquone on day #3	No	Yes
10	Clindamycin/quinine/azithromycin	Azithromycin discontinued on day #5 → changed to azithromycin/atovaquone on day #17 due to hypoglycemia. Patient with prolonged parasitemia	Yes	Yes
11	Clindamycin/Quinine/Atovaquone	Atovaquone discontinued on day #2 → regimen changed to azithromycin/atovaquone on day #3	Yes	Yes

*ID* infectious diseases

### Potential limitations

First, as the infection is rare and sample size is small, some associations may remain undetected. Second, due to retrospective nature of the study, patients’ past medical history may be not complete and additional risk factors for severe babesiosis may be overlooked. Third, effectiveness of azithromycin/atovaquone therapy was also retrospectively assessed and there was no control group in our study. However, no clinical trials are available at this time to compare azithromycin/atovaquone regimen to clindamycin/quinine regimen in patients with severe babesiosis.

## Conclusion

In conclusion, our data indicate that symptomatic babesiosis is uncommon even in endemic regions. Furthermore, these data suggest that recommended treatment regimens for babesia infections are well tolerated and effective even in patients with severe babesiosis.
